# Differences in the Epidemiology of Childhood Infections with Avian Influenza A H7N9 and H5N1 Viruses

**DOI:** 10.1371/journal.pone.0161925

**Published:** 2016-10-03

**Authors:** Jianping Sha, Wei Dong, Shelan Liu, Xiaowen Chen, Na Zhao, Mengyun Luo, Yuanyuan Dong, Zhiruo Zhang

**Affiliations:** 1 Department of Endocrinology, The 421 Hospital of Chinese People’s Liberation Army, Guangzhou, China; 2 School of Public Health, Shanghai Jiaotong University School of Medicine, Shanghai, China; 3 Department of Infectious Diseases, Zhejiang Provincial Centre for Disease Control and Prevention, Hangzhou, China; 4 Department of Senior Cadres, The 421 Hospital of Chinese People’s Liberation Army, Guangzhou, China; 5 National Research Center for Wildlife Born Diseases, Key Lab of Animal Ecology and Conservation Biology, Institute of Zoology, Chinese Academy of Sciences, Beijing, China; The University of Chicago, UNITED STATES

## Abstract

The difference between childhood infections with avian influenza viruses A(H5N1) and A(H7N9) remains an unresolved but critically important question. We compared the epidemiological characteristics of 244 H5N1 and 41 H7N9 childhood cases (<15 years old), as well as the childhood cluster cases of the two viruses. Our findings revealed a higher proportion of H5N1 than H7N9 childhood infections (31.1% vs. 6.4%, *p* = 0.000). However, the two groups did not differ significantly in age (median age: 5.0 vs. 5.5 y, *p* = 0.0651). The proportion of clustered cases was significantly greater among children infected with H5N1 than among children infected with H7N9 [46.7% (71/152) vs. 23.6% (13/55), *p* = 0.005], and most of the childhood cases were identified as secondary cases [46.4% (45/97) vs. 33.3% (10/30), *p* = 0.000]. Mild status accounted for 79.49% and 22.66%, severe status for 17.95% and 2.34%, and fatal cases for 2.56% and 75.00% of the H7N9 and H5N1 childhood infection cases (all *p*<0.05), respectively. The fatality rates for the total, index and secondary childhood cluster cases were 52.86% (37/70), 88.5% (23/26) and 33.33% (15/45), respectively, in the H5N1 group, whereas no fatal H7N9 childhood cluster cases were identified. In conclusion, lower severity and greater transmission were found in the H7N9 childhood cases than in the H5N1 childhood cases.

## Introduction

Avian influenza (AI) refers to a disease caused by influenza type A viruses, which occur naturally among wild aquatic birds but can also infect domestic birds and, to a lesser extent, other animal species and humans [1]. Prior to 2000, only 72 human infections were caused by influenza A viruses from avian or swine sources. After 2000, improvements in surveillance, diagnostic tests, and public awareness resulted in a sharp increase in the number of human cases to 2000. The main strains were the highly pathogenic avian influenza (HPAI) A(H5N1) and the low pathogenic avian influenza (LPAI) A(H7N9) viruses in Asia and swine H3N2v viruses in North America [1]. Almost all of these cases have been epidemiologically linked to close contact with poultry, chiefly chickens or ducks, but human-to-human transmission, although rare, has also been documented [2–5]. The clinical spectrum of AI infections may range from asymptomatic, subclinical, and mild to serious respiratory disease and fatality [3, 6–11]. The clinical outcome is not only linked to viral virulence but also to a host of other factors including age, antiviral treatment and chronic diseases [8, 12–16].

The different avian influenza viruses vary in terms of their epidemic features. Since HPAI H5N1 was first identified in 1997 [17], it has been well-described as an important respiratory pathogen, with the greatest morbidity and rates of hospitalization occurring among children, which is similar to what is observed in adults [13, 18–21]. By contrast, children infected with the novel LPAI H7N9 virus, which was identified in 2013, are typically asymptomatic or have mild symptoms, whereas infections in the total population are severe or even fatal [5, 11, 22–25]. The reasons for this difference are not clear. In this study, we included 41 children infected with H7N9 and 244 children infected with H5N1 (≤15 years old). The childhood index cases and secondary cases belonged to 25 and 55 family clusters, respectively. We aimed to compare key epidemiological variables (disease distribution, severity and transmissibility) of the complete global series of laboratory-confirmed human cases of influenza A H7N9 and H5N1. The results of this comparison will improve our understanding of the different characteristics of these viruses and inform public health control measures for these co-circulating viruses in children.

## Materials and Methods

### Data source

As of June 13, 2016, the laboratory-confirmed cases of avian influenza A H7N9 and H5N1 virus infection are reported to the Zhejiang Center for Disease Control and Prevention (Zhejiang CDC) through the infectious diseases reporting and surveillances systems (the internal data and the internal link), including influenza surveillance, avian influenza surveillance, unexplained pneumonia surveillance, and severe acute respiratory illness (SARI) surveillance. This system owned and maintained routinely by Zhejiang CDC in Hangzhou, China. The other data for H7N9 cases and H5N1 cases outside of Zhejiang Province, were retrieved from the public officially news releases from the China health authority (http://www.nhfpc.gov.cn/jkj/s3578/201312/deab457117644f8ab8a739ea22fdaa71.shtml).

Information regarding all other H5N1 cases was obtained from various publically available sources, including World Health Organization updates (http://www.who.int/csr/don/2005_01_21/en/), news releases from the local health authority (http://www.chp.gov.hk/en/guideline1_year/29/134/332.html), ProMed posts (http://www.promedmail.org/aboutus/publications/), and the published literature (http://www.ncbi.nlm.nih.gov/pubmed/?term=H7N9+AND+CHINA).

As of June 13, 2016, a total of 781 laboratory-confirmed cases of human infection with avian influenza A(H7N9) virus, including at least 313 deaths, have been reported to the WHO. We selected 41 laboratory-confirmed childhood cases of influenza A(H7N9) virus in this study (S1a Fig).

Between 2003 and June 13, 2016, a total of 851 laboratory-confirmed cases of human infection with avian influenza A(H5N1) virus, including 450 deaths, were reported to the WHO from 16 countries (S1b Fig). In this study, we collected 244 cases of children who were infected with avian influenza A(H5N1) virus.

### Case and cluster case definitions

The case definitions, a cluster definition and exposure definitions were established based on ‘the diagnosis and treatment programs of human infections with H7N9 and H5N1 virus’ issued by the National Health and Family Planning Commission of the People’s Republic of China [8].

### Epidemiological and clinical investigation

When a suspected case of H7N9 virus infection was confirmed, the provincial epidemiologists and local public health doctors conducted the initial field investigations using a standard questionnaire to identify the dates, times, frequency and patterns of exposure to poultry and/or other animals, as well as the environments of the birds.

### Data analysis and statistics

All maps were generated using ARCGIS 10.2 software (http://resources.arcgis.com/en/home/). All statistical analyses were conducted using the Statistical Analysis System, version 9.2 (SAS Institute, Cary, NC, USA). Quantitative measurements are presented as median values, and qualitative measurements are presented as relative and absolute frequencies. Analysis of variance (F test) was applied to the measured data. Chi-square tests (*x*^*2*^) were used to compare the distributions of the different variables of qualitative measurements between the two groups. All reported p values are two-sided and were considered statistically significant at 0.05.

## Results

### Epidemiological comparison

#### Disease distribution

**Fig 1:**
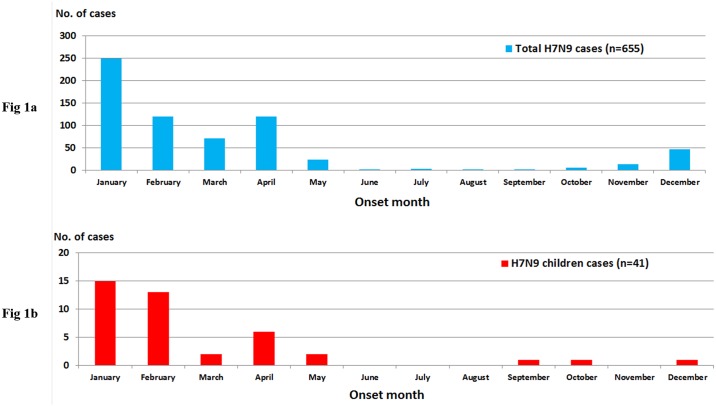
Monthly distribution curve for the total confirmed cases and childhood cases of avian influenza A(H7N9) between February 18, 2013, and June 13, 2016. Notes: Fig 1a: Total cases (N = 655); Fig 1b: Childhood cases (N = 41).

**Fig 2:**
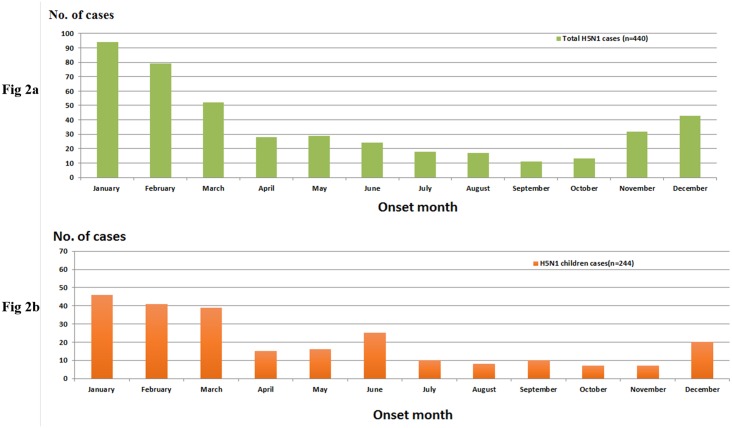
Monthly distribution curve of confirmed avian influenza A(H5N1) cases in the overall global population and in children between 2006 and June 13, 2016. Notes: Fig 2a: Total cases (N = 440); Fig 2b: Childhood cases (N = 244).

Based on the available data for the global totals of 781 H7N9 and 851 laboratory-confirmed cases, we analyzed 644 confirmed H7N9 (including 41 childhood cases) and 784 H5N1 cases (including 244 childhood cases), respectively, for analysis in this study. The proportion of childhood cases of H7N9 infection was much smaller than that of H5N1 [6.4% (41/644) vs. 31.1% (244/784), p = 0.000]. The childhood cases were distributed in 11/16 and 10/16 areas/countries in the H7N9 and H5N1 groups, respectively (S1a and S1b Fig). The seasonal distribution of H5N1 and H7N9 coincide well with the childhood and overall cases, which occurred from December to February of each year in China (Figs 1a, 1b, 2a and 2b).

**Fig 3:**
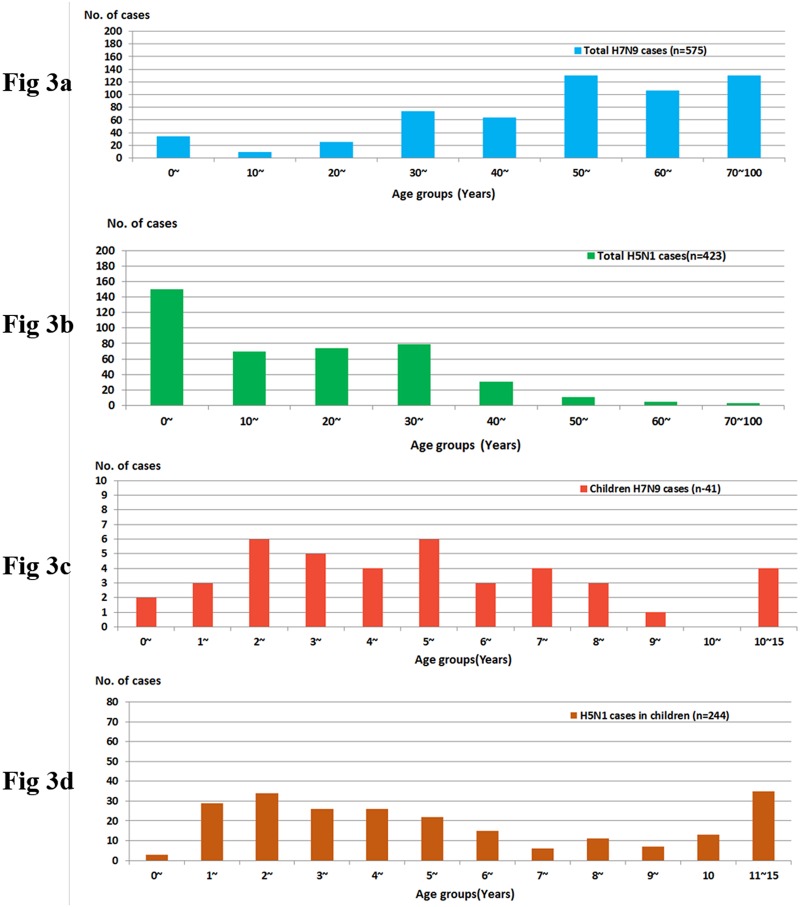
Age distribution for the total and childhood cases of infection with avian influenza H7N9 and H5N1 viruses. Notes: Fig 3a: H7N9 total cases (n = 575); Fig 3b: H5N1 total cases (n = 423); Fig 3c: H7N9 childhood cases; Fig 3d: H5N1 childhood cases.

The ages of the H7N9 cases are unusual compared with those of the H5N1 cases. For the total cases, the median age of the H7N9 group was 53 (0.4~91) years old, which was much older than that of the H5N1 group [20.5 (0.7–75) years old, *p* = 0.000]. The predominant age was 50 years old and 0 years old in the H7N9 and H5N1 groups, respectively (Fig 3a and 3c). By contrast, for the childhood cases, the median age in the H7N9 group was 5.0 (0.4–15) years old, which was not significantly different from that of the H5N1 group [5.5 (0.8–15) years old, *p* = 0.065] (Fig 3b and 3d). The predominant age was 2 years old for both groups.

**Fig 4:**
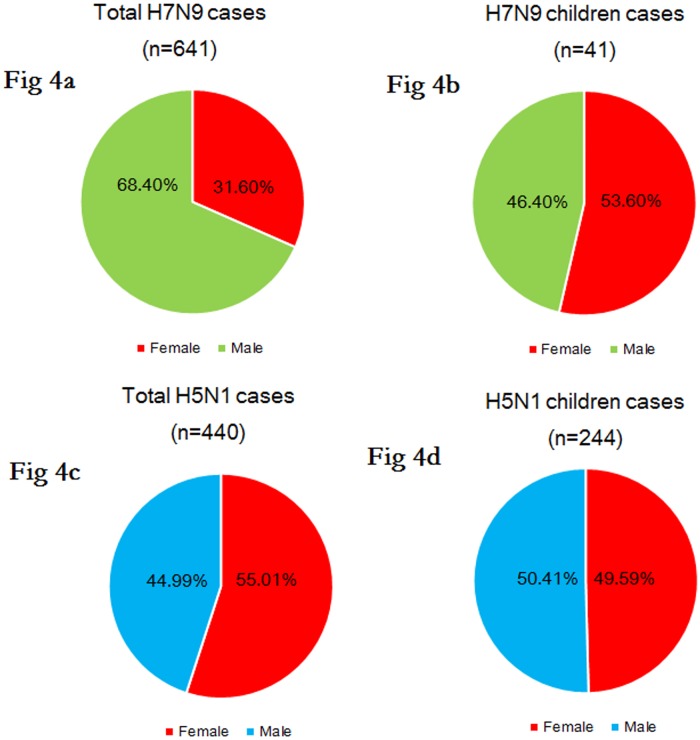
Sex distribution of the confirmed total (n = 641) and childhood (n = 41) avian influenza A(H7N9) cases and of the global total (n = 440) and childhood (n = 244) confirmed H5N1 cases. Notes: Fig 4a: H7N9 total cases; Fig 4b: H7N9 childhood cases; Fig 4c: H5N1 total cases; Fig 4d: H5N1 childhood cases.

For the total cases, among 641 H7N9 cases, male cases were twice as common as female cases [female 31.60% versus male 68.40%] (Fig 4a). By contrast, a sex distribution balance was identified in 440 H5N1 cases [female 55.01% versus male 44.99%, *p* = 0.000]. (Fig 4c). For childhood cases, the sex differences were more evenly distributed in the H7N9 (female 53.60% versus male 46.40%) and H5N1 groups [female 49.59% versus male 50.41%, *p* = 0.630] (Fig 4b and 4d).

### Exposure history

For various types of poultry exposure, exposure was less commonly reported in H7N9 cases than in H5N1 cases (*p* = 0.000); however, most of these differences were not statistically significant between the total and childhood H5N1 cases (*p*>0.05). The number of visits to live bird markets and human-to-human transmission were much greater for the childhood cases of H7N9 than H5N1 (*p =* 0.000). However, the history of exposure to sick or dead poultry was more common for the childhood cases of H5N1 than H7N9 (*p =* 0.000). There were no differences in exposure to backyard poultry between the two groups of children (*p =* 0.061) (Table 1).

**Table 1:** Comparison of the exposure history in childhood and total cases infected with the avian influenza H7N9 and H5N1 viruses.

Exposure history	H7N9 groups			H5N1 groups			*p3*	*p4*
	Total (n = 440)	Child (n = 41)	*p1*	Total (n = 412)	Child (n = 196)	*p2*		
**Any exposure to poultry**	290 (65.9%)	26 (63.4%)	0.748	360 (86.12%)	177 (90.31%)	0.293	0.000	0.000
**Occupational exposure to live poultry**	28 (6.4%)	0 (0.0%)	0.096	4 (0.96%)	0 (0.0%)	0.166	0.000	-
**Visit LBMs**	213 (48.4%)	17 (41.5%)	0.394	29 (6.94%)	15 (7.65%)	0.785	0.000	0.000
**Exposure to sick or dead poultry**	8 (1.8%)	3 (7.3%)	0.024	213 (50.96%)	99 (50.51%)	0.784	0.000	0.000
**Exposure to backyard poultry**	84 (19.1%)	11 (26.8%)	0.234	55 (13.16%)	29 (14.80%)	0.629	0.023	0.061
**Human case contact**	5 (1.1%)	7 (17.1%)	0.000	4 (0.72%)	2 (1.02%)	0.954	0.813	0.000

Note: LBMs = live bird markets

*p1*: comparison of the total and childhood cases infected with H7N9;

*p2*: comparison of the total and childhood cases infected with H5N1;

*p3*: comparison of the total cases infected with H7N9 and H5N1;

*p4*: p3: comparison of the childhood cases infected with H7N9 and H5N1.

### Childhood cases in clusters

**H7N9 family clusters. Disease distribution:** As of June 13, 2016, 781 cases (including 41 children) of H7N9 were recognized globally, including 25 clusters of cases. Cluster cases account for approximately 7.0% (55/781) of the total cases. Childhood cases (<15 years old) were identified in 23.6% (13/55) of the family cluster cases, of which three cases (accounting for 12% [3/25]) were index cases and 10 cases (accounting for 33.3% [10/30]) were secondary cases (Table 2).

**Table 2:** Comparison of family clusters infected with the avian influenza H7N9 and H5N1 viruses.

Characteristics	H7N9 clusters (N = 25)						H5N1 clusters (N = 55)					
	Total		Index cases		Secondary cases		Total		Index cases		Secondary cases	
	Overall (n = 55)	Children (n = 13)	Overall (n = 25)	Children (n = 3)	Overall (n = 30)	Children (n = 10)	Overall (n = 152)	Children (n = 71)	Overall (n = 55)	Children (n = 26)	Overall (n = 97)	Children (n = 45)
**Percent (Cluster cases/total cases)**	7.0% (55/781)	23.6% (13/55)	45.5% (25/55)	12% (3/25)	54.5% (30/55)	33.3% (10/30)	19.4% (152/784)	46.71% (71/152)	36.2% (55/152)	47.3% (26/55)	63.82% (97/152)	46.4% (45/97)
**Median Age (Years)**	40 (0.75~87)	4.0 (0.75~8)	40 (0.75~77)	2.25 (0.75~5)	34 (1.8~87)	4.5 (1.8~8)	19 (0.3~80)	8.0 (0.3~15)	20.8 (5~69)	9.84 (5~15)	18.3 (0.3~80)	7.0 (0.3~15)
**Female: male**	0.7:1.0	2.25:1.0	1.0:2.0	1.9:1.0	0.9:1.0	2.3:1.0	1.1:1.0	1.5:1.0	1.6:1.0	4.5:1.0	1.0:0.9	0.9:1.0
**Case-fatality rate (%)**	29.09 (16/55)	(0/13)	40 (10/25)	0 (0/3)	20 (6/30)	0 (0/10)	57.89% (88/152)	52.86% (37/70)	80.00% (44/55)	88.5% (23/26)	44.33% (43/97)	33.33% (15/45)
**Female-related CFR**	17.4% (4/23)	(0/13)	22.2% (2/9)	0 (0/2)	14.3% (2/14)	0 (0/7)	63.75% (51/80)	57.14% (24/42)	85.3% (29/34)	80.95% (17/21)	48.89% (22/45)	33.3% (7/21)
**Age-related CFR**												
**0~4**	-	0.00 (0/10)	-	0.00 (0/3)	-	0.00 (0/7)	36% (9/25)		-	0.00% (0/0)	-	31.25% (5/16)
**5 ~9**	-	0.00 (0/3)	-	0.00 (0/0)	-	0.00 (0/3)	48% (12/25)		-	71.43% (10/14)	-	21.43% (3/14)
**10~14**	-	0.00 (0/0)	-	0.00 (0/0)	-	0.00 (0/0)	80% (16/20)		-	100% (12/12)	-	50.00% (7/14)
**Over 15 years**	38.10 (16/42)	-	45.45 (10/22)	-	30.00 (6/20)	-	64.10% (50/78)		75.86% (22/29)	-	57.14% (28/49)	-

Cluster cases were reported in 10 of the 17 areas/countries infected with the H7N9 virus. (Table 2). H7N9 cluster cases were reported between 2013 and 2016 and peaked in 2014. Cluster cases were mainly identified between November and May of each year and peaked between January and February, with 50% of the cluster cases identified in January (Table 2).

The mean age of the cluster-associated H7N9 cases was 40 (0.75–87) years, compared with 4.0 years for the childhood cases (0.75–8 years). Among the 25 index cases, the average age was 40 (0.75~77) years. Of these, the childhood index cases ranged from 0.75 to 5 years old, with an average of age of 2.25 years. By contrast, the median ages were 34 (1.8~87) and 4.5 (1.8–8) years old for the overall and childhood secondary cases, respectively (Table 2).

Among the cluster-associated childhood cases of H7N9, 69.23% (9/13) were male, whereas 30.77% were female (4/13). Among the 25 index cases, the ratio of females to males was 1:1.9; however, the female to male ratio was 2:1 in the three childhood cases. Among the 30 secondary cases, the female to male ratio was 0.9:1.0, but the ratio was 2:3 for the secondary childhood cases (Table 2).

**Case fatality rate:** The case fatality rate (CFR) in the total clustered cases was 29.09% (16/55), which was much lower than the index cases [40% (10/25)] but was slightly higher than the secondary cases [(20% (6/30)]. For the cluster-associated cases, the CFR was significantly higher for the male cases than the female cases: 17.4% (4/23) for females and 37.5% (12/32) for male cluster cases. Among the 25 index cases, the CFR was 22.2% (2/9) for females and 50% (8/16) for males. Among the 30 secondary cases, the CFR was 14.3% (2/14) for females and 25% (4/16) for males (Table 2). None of the children from the cluster cases died of the H7N9 influenza virus.

**Cluster size:** For the H7N9 clusters, the average cluster size was 2.2 cases (range: 2–4); 21 of 25 (84%) family clusters involved two members, 12% (3/25) of clusters involved three members, and only one cluster [4%(1/25)] involved four family members. Among the 30 secondary cases, 40% (12/30) of the cluster cases occurred among blood-related family members, suggesting a possible genetic susceptibility (S1 Table).

**H5N1 family clusters. Disease Distribution:** As of June 13, 2016, 851 confirmed human cases of H5N1 virus infection were identified and reported to the WHO. Of these, 55 clusters involving 152 cases with at least two epidemiologically linked cases were identified (118 cases confirmed and 34 probable), accounting for approximately 17.86% (152/851) of the total cases. Interestingly, childhood cases (<15 years old) were identified in 46.7% (71/152) of these cluster cases, of which 26 children were identified in 47.3% (26/55) of the index cases. However, 45 children were identified in 46.4% (45/97) of the secondary cases (Table 2).

The percentage of all cases occurring in clusters was relatively stable in 11 out of the 16 reported areas/countries worldwide (Table 2).

Cluster cases were identified throughout the year, and 52.7% of the cluster cases were reported in December, January and February (Table 2).

The mean age of all cluster cases was 19 (0.3–80), compared with 22 (1–75) years old for sporadic cases. The mean age was 8.0 (0.3–15) years old for 71 childhood cluster cases. However, the average age was 20.8 (5–69) years old for 55 of the index cases, of which the mean age was 9.84 (5–15) years old among the 26 childhood cases. For the secondary cases in the overall population, the median age was 18.3 (0.3–80) years old. By contrast, the median age was 7.0 (0.3–15) years old for the childhood secondary cases (Table 2).

Among the overall cluster-associated cases, 52.6% (80/152) were female, and 47.4% (72/152) were male. Of these, there was a female and male distribution of 59.15% (42/71) and 40.85% (29/71) in the 71 childhood cases, respectively. In the 55 index cases, the female versus male distribution was 61.82% (34/55) versus 38.18% (21/55), respectively. Among the 26 childhood index cases, 80.8% (21/26) versus 19.2% (5/26) were identified in the female and male populations, respectively. For the 97 secondary cases, the female versus male case ratio was 46.39% (45/97) and 53.61% (52/97), respectively. For the 45 secondary childhood cases, female versus male cases accounted for 46.7% (21/45) versus 53.3% (24/45) of the secondary cases, respectively (Table 2).

**CFR:** The case fatality rate for the total clustered cases was 57.89% (88/152), which was much lower than the index cases [80.00% (44/55)] but slightly higher than the secondary cases [44.33% (43/97)]. The same results were identified in the childhood cluster cases [52.86% (37/70) vs. 88.5% (23/26) vs. 33.33% (15/45) for the total, index and secondary cases, respectively] (Table 2).

For the cluster-associated cases, the highest CFRs were both identified in the 10-15-year-old group among the total cluster [(80%, 16/20)], index [100% (12/12)] and secondary cases [50.00% (7/14)] (Table 2).

For the cluster-associated cases, the CFR in the female cases was significantly higher than that in the male cases: 57.14% (24/42) for females versus 46.43% (43/28) for males in the total clustered childhood cases. However, there were no gender differences in the secondary cases.

Among the cluster-associated cases, death was associated with the case order and occurred in 44/55 (80%) primary or co-primary cases, 30/55 (54.55%) secondary cases, 8/19 (42.11%) tertiary cases, 3/7 (42.86%) quaternary cases, 1/4 (25.00%) fifth-order cases, 0/0 (0%) for sixth-order cases, and 1/3 (33.33%) for both seventh- and eighth-order cases (Table 2).

**Cluster size:** The average cluster size was 2.8 cases (range: 2–10) and remained stable by country. Approximately 65.45%

Of the family clusters involved 2 members. Among the 97 secondary cases, 89.70% (87/97) of the cluster cases occurred among blood-related family members, suggesting a possible genetic susceptibility (S1 Table).

### Clinical comparison

#### Median days

The median days from onset to admission was much shorter for the childhood H7N9 than for the H5N1 cases [1 day vs. 4 days, *p* = 0.000].

The median number of days from disease onset to antivirus treatment was shorter in H7N9 childhood cases than total H7N9 cases and child H5N1 cases [1 day vs. 4.5 days vs. 5.0 days, respectively, *p* = 0.000]. However, the median number of days from onset to treatment was clearly longer for fatalities than for survivors [7 days vs. 4 days, *p* = 0.001].

The same results were obtained for the median number of days from onset to confirmation.

The median days from onset to death was longer in the H7N9 than in the H5N1 group [13 days vs. 10 days, *p* = 0.005].

The median number of days from disease onset to discharge was shorter in children infected with the H7N9 than in children infected with the H5N1 virus [8.5 days vs. 11 days, *p* = 0.035] (S2 Table).

Among the cluster-associated cases infected with avian H5N1 virus, the average number of days from Case 2 to index case onset was 5.0 (0–16) days; from Case 3 to index case onset was 10.0 (0–24) days; from Case 4 to index case onset was 14 (0–23) days; from Case 5 to index case onset was 11.0 (0–23) days; from Case 6 to index case onset was 19.0 (15–23) days; and from Case 8 to index case onset was 21 (n = 1) days (S2 Fig).

Among the 25 family clusters with H7N9, the average number of days from Case 2 to index case onset was 8.0 (3–17) days and from Case 3 to index case onset was 10.0 (8–12) days (S2 Fig).

#### Clinical severity

**Fig 5:**
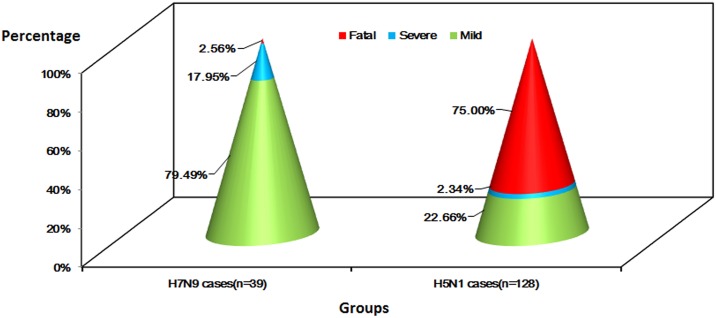
Clinical severity of confirmed avian influenza A(H7N9) cases in children (n = 39) and confirmed H5N1 cases in children (n = 128) worldwide.

The clinical spectrum was analyzed in 39 confirmed childhood cases of H7N9 and 128 confirmed childhood cases of H5N1; mild cases accounted for 79.49% (H7N9) versus 22.66% (H5N1), severe cases accounted for 17.95% (H7N9) versus 2.34% (H5N1), and fatalities accounted for 2.56% (H7N9) versus 75.00% (H5N1) of the cases (Fig 5).

## Discussion

One epidemiological similarity of HPAV H5N1 and LPHV H7N9 is that are both derived from poultry or related to a poultry environment [8, 21, 26]. Another similarity is their seasonal distribution, which coincides well with the anticipated annual epidemic curves in the northern hemisphere of seasonal human influenza from November through April [1]. The third similarity is that both avian viruses exact a disproportionate health toll on children compared with adults. The characterized H5N1 virus carried a lower mortality rate in children (52.86%) and higher mortality rate in adults aged >15 years (64.10%) in this large case series. Similar results were obtained for the H7N9 groups (CRR of 0% vs. 38.10% in children and adults, respectively). The less mature immune systems of younger children might mount a response that is less harmful to the host [27]. However, the epidemiological features of these two emerging infections in children differ from those in adults in some meaningful ways. These difference depend on age and gender, underlying diseases, exposure history, time to treatment initiation, and reported country, etc. [7, 27–30].

First, childhood cases of infection with the two viruses have a geographical distribution that is similar to the overall cases. H5N1 cases occur mostly in Asia and Africa [28, 29], but a significantly higher number of H7N9 cases occur in Asia, predominantly in China. The differing patterns induced by the two viruses may represent breeding, animal-human contact behavior, surveillance, and control efforts, among others [31–33]. Second, the age and sex characteristics of the childhood cases are unusual compared with the overall cases infected with these two emerging viruses [9]. By contrast, H5N1 cases exhibit a more equal sex distribution in the overall populations. We found a much lower proportion of childhood cases infected with H7N9 among the total cases (6.4%) than in the H5N1 groups (31.1%). However, the age and sex findings were consistent for the children infected with the H7N9 and H5N1 viruses. These phenomena are not understood but have been attributed to the routes of exposure, cultural practices and underlying conditions [14, 25]. Another important factor in determining if a novel virus will emerge to cause a pandemic is the degree of immunity to the virus in the population [34].

Generally, direct avian-to-human H5N1 and H7N9 virus transmission is the predominant means of human infection [21]. H5N1 circulates in wild birds and infects poultry in backyards and small farms in rural areas [35]. By contrast, the 2013 H7N9 in China appears to have been generated relatively recently through reassortment and has not been found to any significant degree in rural farms [1]. In the present study, a history of exposure to sick or dead poultry was more common for H5N1 childhood cases than for H7N9 cases (50.51% vs. 7.3%), as would be expected given the highly pathogenic phenotype of H5N1 in poultry and the low pathogenic phenotype of H7N9 [36]. Most childhood patients have acquired A(H5N1) infection from poultry raised inside or outside their houses after playing with or holding diseased or dead poultry [12, 35, 37, 38]. Thus, handling sick or dead poultry is the most commonly recognized risk factor for child cases of infection with H5N1 avian influenza [39]. This finding will contribute to the early ascertainment, investigation and isolation of childhood cases of human infection with H5N1 virus and, consequently, decreased transmissibility. In addition, our findings indicate that childhood cases in urban settings had visited live bird markets (LBMs) more frequently prior to illness onset than childhood cases of H5N1 virus (41.5% vs. 7.65%). These results indicate that contamination of LBMs and bird-to-bird transmission of H7N9 in these markets may be the primary initial mechanisms for amplifying transmission of the virus and represent a focus for the implementation of control measures against H7N9 virus infection in children [1, 21]. However, control of the exposure source will be challenging because the H7N9 virus began circulating silently in poultry markets and infected birds show no symptoms, contributing to the higher transmission and potential pandemic risk for childhood infections with H7N9 than for childhood infections with H5N1 [1].

The well-described clusters of cases with H5N1 and H7N9 support limited, nonsustained transmission without any super spreaders [40, 41]. The calculated transmission dynamics model also did not support person-to-person transmission. The R0, a measure of transmission potential, was 0.27, 0.1 and 1.7–2.1 for H5N1, H7N9 and 2009H1N1pdm, respectively [42]. This result showed much higher transmission of 2009H1N1pdm than of the H7N9 and H5N1 avian viruses. Our findings also support the observation that human-to-human transmission leading to a potential pandemic risk may be greater for H7N9 than H5N1. There were no significant differences in the transmission of the two viruses between the children and the overall cases.

For the total cases, it is well known that the CFR in H7N9 is much lower than that in the H5N1 groups, but it was higher than the cases of seasonal influenza in China [43]. By contrast, for the childhood cases, the high fatality (75.00%) associated with the H5N1 infections is strikingly different from all outbreaks of human childhood cases of H7N9 infection. Only one death has been reported during an outbreak of H7N9. This high CFR of H5N1 is probably a consequence of many mild and even asymptomatic infections that have not been identified because of insufficient public health resources in areas with high infection rates. However, the children infected with H7N9 viruses were identified through sentinel surveillance of Influenza-like Illness (ILI) or were traced by contact through a family cluster [11]. These cases could be identified and admitted, and antivirus treatment could be initiated early so that they presented mild symptoms. Those children with a mild case of H7N9 remained a potential infection source of avian virus. Thus, there is an urgent need to develop a rapid, sensitive, and specific diagnostic test to confirm H7N9 infection as early as possible [44, 45]. In the clustered cases infected with the two viruses, the CFR in the secondary cases was much lower than that in the index cases. This finding was similar to that of Qin Y et al. [36]. For the H5N1 clusters, there were no significant differences in the CFR of the index cases between the total and childhood cases; however, in the secondary cases, the CFR in the child cases was much lower than that in the total cases. An alternative explanation is that the children in the secondary cases were much younger and had a lower rate of underlying diseases than the total secondary cases [10, 46]. Importantly, the clinical period (from onset to discharge) was much shorter for the total H5N1 cases than the H7N9 groups. This result shows that H5N1 infections were much more severe than H7N9 infections due to higher respiratory tract viral loads. In addition, this difference was not only related to the detection capability and timeliness of medical seeking behavior but also to medical care management.

In conclusion, our findings suggest that the disease distribution of laboratory-confirmed childhood H7N9 cases is not biased compared with all H5N1 cases. However, our research suggests that these two viruses possess quite different risk profiles. First, disease transmission in childhood cases of H7N9 was much higher than that in the H5N1 groups. Second, the disease severity in the childhood cases of H7N9 was significantly reduced in comparison to the children who were infected with the H5N1 virus. Moreover, the severity of the secondary childhood cases was slightly lower than that of the total cases. This difference was attributed to the discovery methods, the time of antivirus treatment initiation, and the underlying conditions. These factors will make the detection and monitoring of changes in viruses more challenging in the future.

## Supporting Information

S1 Fig Geographic distribution of confirmed cases of H7N9 and H5N1 in the overall and child populations as of June 13, 2016. Notes: 1a: Total H7N9 cases (N = 781) and child cases (n = 41); 1b: Total H5N1 cases (N = 851) and child cases (n = 244).
(PPTX)

S2 Fig Timeline from the onset of primary to secondary cases from the clustered cases caused by H7N9 and H5N1 viruses. (PPTX)

S1 Table Comparison of the size of family clusters infected with avian influenza H7N9 and H5N1 virus. (DOCX)

S2 Table Comparison of the median days from onset to outcome in the H5N1 and H7N9 infections. (DOCX)

## Abbreviations

AI: Avian influenza
CFR: Case fatality rate
HPAI: Highly pathogenic avian influenza
ICU: Intensive care unit
ILI: Influenza-like illness
LPM: Live poultry market
LPAI: Low pathogenic avian influenza
