# Supplementary figures and images for: Differences in the Epidemiology of Childhood Infections with Avian Influenza A H7N9 and H5N1 Viruses
Source: PLoS One. 2016 Oct 3;11(10):e0161925. doi: 10.1371/journal.pone.0161925 (PMC5047462; doi:10.1371/journal.pone.0161925)

## Slide 1
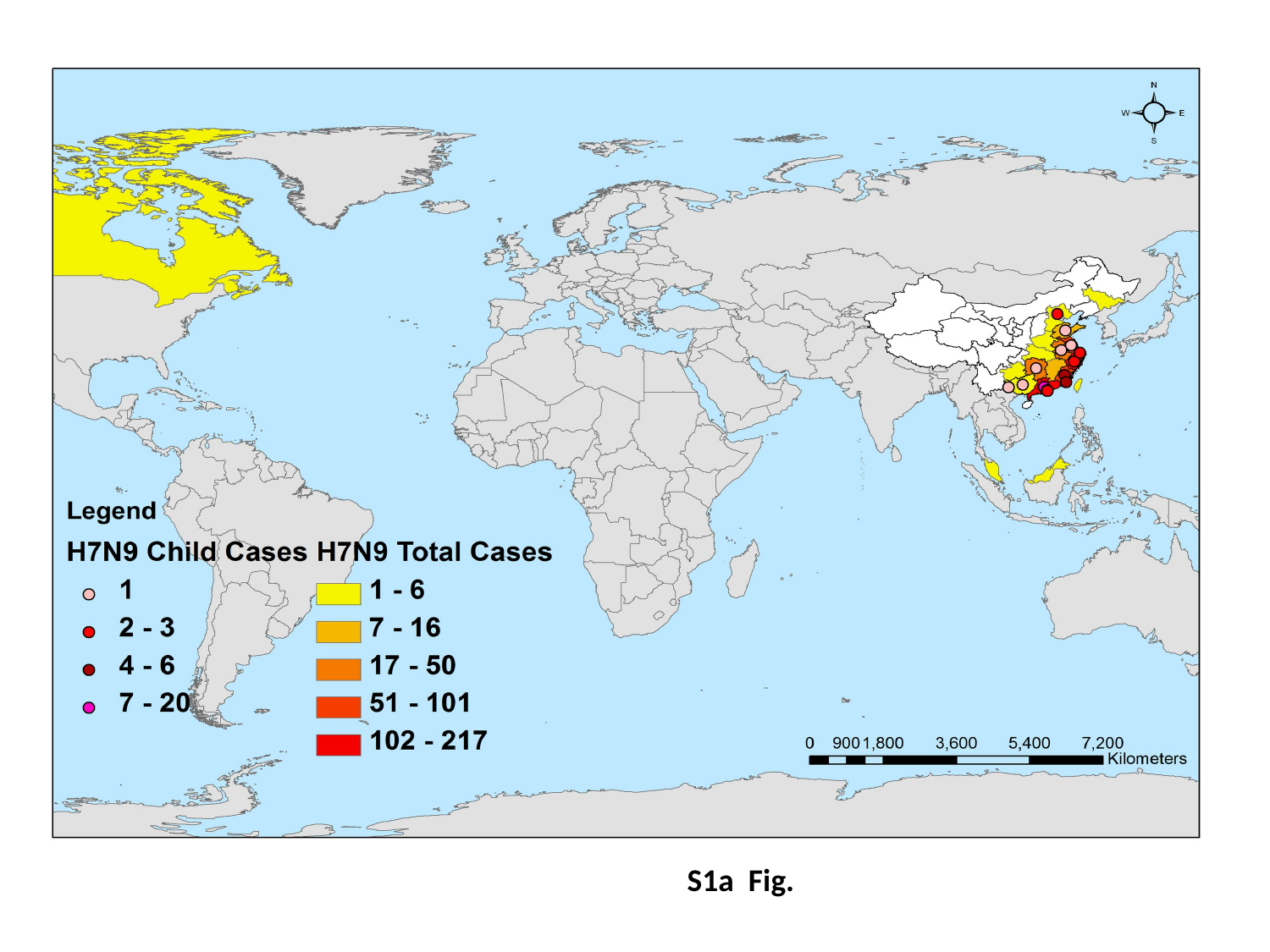

S1a Fig.

## Slide 2
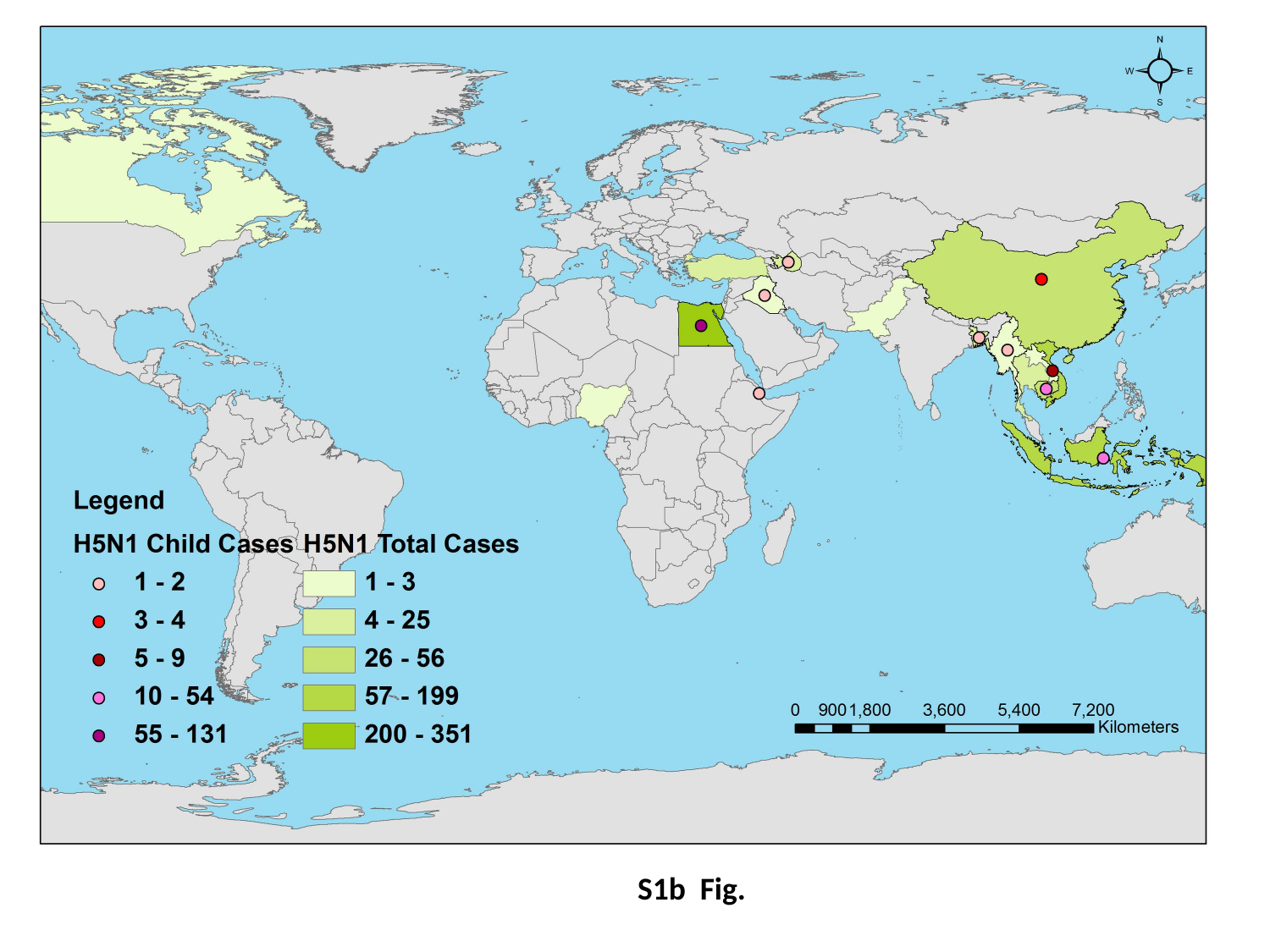

S1b Fig.

Supplement: S1 Fig — Notes: 1a: Total H7N9 cases (N = 781) and child cases (n = 41); 1b: Total H5N1 cases (N = 851) and child cases (n = 244). (PPTX) [file pone.0161925.s001.pptx]

## Slide 1
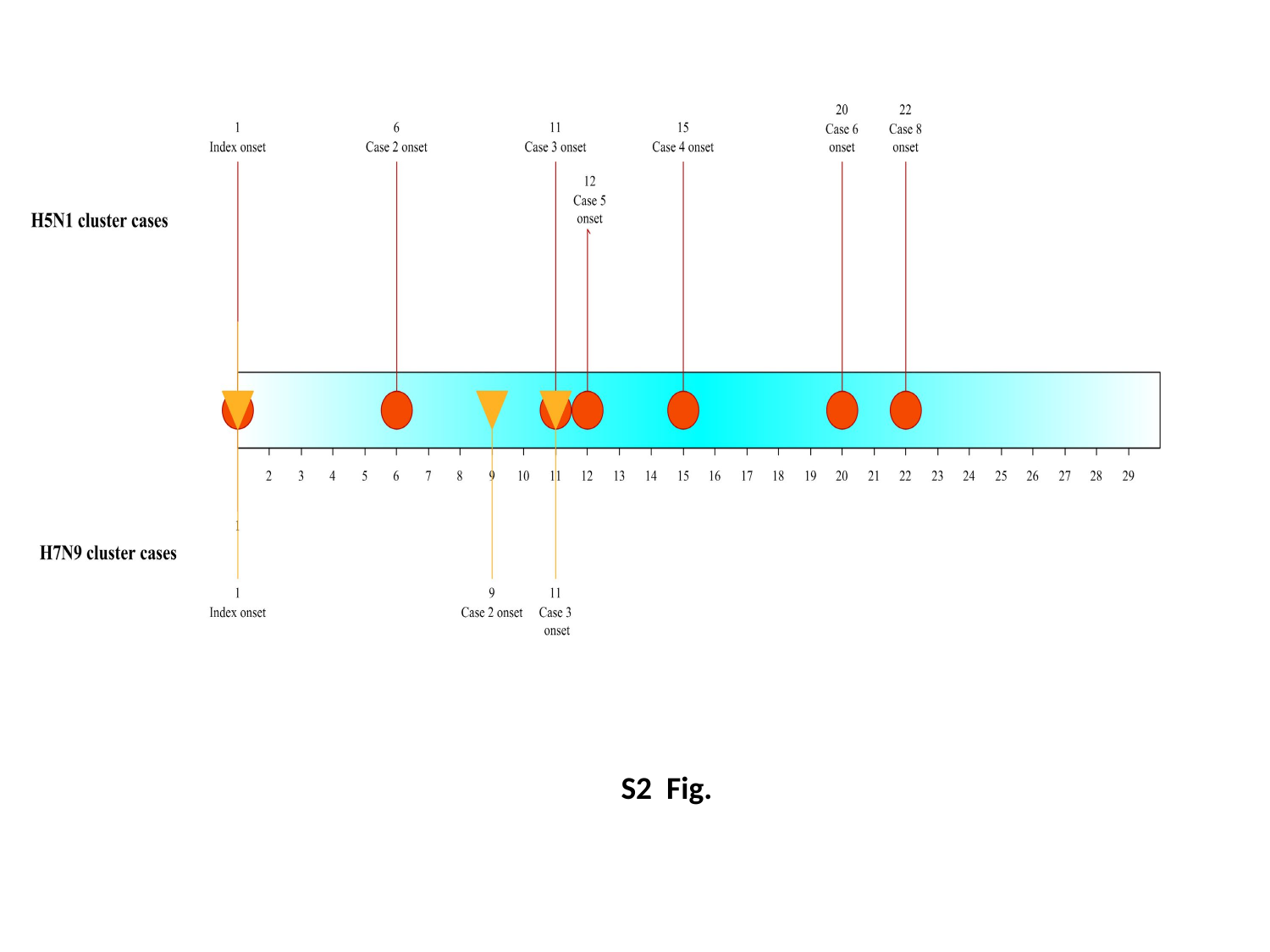

S2 Fig.

Supplement: S2 Fig — (PPTX) [file pone.0161925.s002.pptx]
