# Supplementary material for: Differences in the Epidemiology of Childhood Infections with Avian Influenza A H7N9 and H5N1 Viruses
Source: PLoS One. 2016 Oct 3;11(10):e0161925. doi: 10.1371/journal.pone.0161925 (PMC5047462; doi:10.1371/journal.pone.0161925)
Supplement: S1 Table — (DOCX) [file pone.0161925.s003.docx]

**S1 Table**

| **Groups** | **H7N9 clusters**  **(n=24)** | | **H5N1 clusters**  **(n=55)** | |
| --- | --- | --- | --- | --- |
| **No. of cases per cluster** | **No. of clusters (%)** | **No. of involved cases (%)** | **No. of clusters (%)** | **No. of involved cases (%)** |
| **2 cases** | 20 (83.33%) | 40 (75.47%) | 36 (65.45%) | 72 (47.37%) |
| **3 cases** | 3 (12.5%) | 9 (16.98%) | 11 (20.00%) | 33 (21.71%) |
| **4 cases** | 1 (4.17%) | 4 (7.55%) | 4 (7.27%)) | 16 (10.53%) |
| **5 cases** | - | - | 1 (3.64%) | 5 (3.29%) |
| **6 cases** | - | - | 0 (0.00%) | 0 (0.00%) |
| **7 cases** | - | - | 0 (0.00%) | 0 (0.00%) |
| **8 cases** | - | - | 2 (3.64%) | 16 (10.53%) |
| **10 cases** | - | - | 1 (1.82%) | 10 (6.58%) |
| **Average size** | 2.2 (2~4) | - | 2.8 (2~10) |  |
| **Total** | **24** | **53** | **55** | **152** |
