# Supplementary material for: Differences in the Epidemiology of Childhood Infections with Avian Influenza A H7N9 and H5N1 Viruses
Source: PLoS One. 2016 Oct 3;11(10):e0161925. doi: 10.1371/journal.pone.0161925 (PMC5047462; doi:10.1371/journal.pone.0161925)
Supplement: S2 Table — (DOCX) [file pone.0161925.s004.docx]

**S2 Table**

| **Groups** | **H7N9** | | | | | | **H5N1** | | | | | | |
| --- | --- | --- | --- | --- | --- | --- | --- | --- | --- | --- | --- | --- | --- |
|  | **Total cases** | | **Clusters cases** | |  | | **Total cases** | | **Clusters cases** | | | | |
|  | **Overall**  **(n=644)** | **Children**  **(n=41)** | **Overall**  **(n=53)** | **Children**  **(n=13)** | **Deaths (n=14)** | **Survivors**  **(n=39)** | **Overall**  **(n=397)** | **Children**  **(n=194)** | **Overall**  **(n=152)** | **Children**  **(n=71)** | **Deaths (n=88)** | **Survivors**  **(n=55)** |  |
| **Incubation** | 3.3  (1.4~5.7) | 3.3  (1~7) | 4.0  (0~13) | 5.0  (3~6) | 5.0  (1~13) | 3.0  (0~6) | 2~5  (0~7) | 3.0  (0~7) | 4.5  (1~9) | 3.0  (1~4) | 3.0  (1~6) | 5.5  (3~9) |  |
| **From onset to be admitted** | 5.0 (0~15) | 1.0  (0~8) | 4.0 (0~14) | 4.0 (0~14) | 4.0 (0~10) | 4.0 (0~14) | 4.0  (0~17) | 4.0  (0~25) | 5.0  (0~14) | 5.0 (0~10) | 4.0 (1~14) | 5.0  (0~10) |  |
| **From onset to antivirus** | 5.0（0~15） | 1.0  (0~3) | 5.0 (0~14) | 3.0  (0~14) | 7.0  (1~12) | 4.0  (0~14) | 4.0  (0~16) | 4.5 (0~25) | 6.0 (0~17) | 4.0  (0~12) | 7.0  (5~12) | 5.0  (1~17) |  |
| **From onset to be confirmed** | 8.0 (0~67) | 3.0  (1~8) | 10.0 (0~57) | 4.0  (2~14) | 11  (7~23) | 6.0  (0~16) | 6.5  (2~15) | 6.3 (3~15) | 10  (2-18) | 10  (3-17) | 11  (6-18) | 9  (2-17) |  |
| **From onset to death** | 16  (3~61) | 13  (1 death) | 28  (7~85) | No death | 28  (7~85) | - | 10  (2~24) | 10 (2~25) | 8.0  (2~22) | 8.0  (4~11) | 8.0  (2~22) | - |  |
| **From onset to be discharged** | 35  (4~138) | 8.5 (2~18) | 16  (6~40) | 12  (6~13) | - | 16  (6~40) | 15  (7~32) | 11 (7~20) | 19  (9~33) | 20  (12~33) | - | 19  (9~33) |  |
